# Dynamic Selection Techniques for Detecting GPS Spoofing Attacks on UAVs

**DOI:** 10.3390/s22020662

**Published:** 2022-01-15

**Authors:** Tala Talaei Khoei, Shereen Ismail, Naima Kaabouch

**Affiliations:** School of Electrical Engineering and Computer Science, University of North Dakota, Grand Forks, ND 58202, USA; shereen.ismail@ndus.edu (S.I.); naima.kaabouch@ndus.edu (N.K.)

**Keywords:** unmanned aerial vehicles, global positioning system, GPS spoofing attacks, detection techniques, machine learning, dynamic selection, hyperparameter tuning

## Abstract

Unmanned aerial vehicles are prone to several cyber-attacks, including Global Positioning System spoofing. Several techniques have been proposed for detecting such attacks. However, the recurrence and frequent Global Positioning System spoofing incidents show a need for effective security solutions to protect unmanned aerial vehicles. In this paper, we propose two dynamic selection techniques, Metric Optimized Dynamic selector and Weighted Metric Optimized Dynamic selector, which identify the most effective classifier for the detection of such attacks. We develop a one-stage ensemble feature selection method to identify and discard the correlated and low importance features from the dataset. We implement the proposed techniques using ten machine-learning models and compare their performance in terms of four evaluation metrics: accuracy, probability of detection, probability of false alarm, probability of misdetection, and processing time. The proposed techniques dynamically choose the classifier with the best results for detecting attacks. The results indicate that the proposed dynamic techniques outperform the existing ensemble models with an accuracy of 99.6%, a probability of detection of 98.9%, a probability of false alarm of 1.56%, a probability of misdetection of 1.09%, and a processing time of 1.24 s.

## 1. Introduction

The use of unmanned aerial vehicles (UAVs) in military and civilian applications has exponentially increased over the last decade. Military applications include inspection and patrol, surveillance, reconnaissance, area mapping, and strike and rescue missions. Civilian applications include multimedia shooting, agricultural monitoring, meteorological monitoring, disaster detection, traffic control, cargo transportation, delivery services, and emergency rescue. Middle and long-distance applications rely heavily on Global Positioning Systems (GPSs) for navigation and precise positioning tasks [1].

Huge technical advances in the design, control, and automation have been made over the last two decades; however, the security aspect of UAVs has been largely overlooked [2]. UAVs can be subject to several cyber-attacks, such as GPS spoofing and jamming, which can impact the safety of civilians and airspace. Several UAV security incidents were reported during warfare and conflicts in Iran, Ukraine, and Iraq. During these attacks, malicious users transmitted fake GPS signals with incorrect positional and timing data that could be easily detected, resulting in erroneous navigation. These signals are similar to those from by satellites and are indistinguishable from authentic GPS signals.

A number of techniques have been proposed to detect GPS spoofing attacks. These methods can be classified into three categories [3]: cryptography-based, signal processing methods, and external UAV characteristics. Cryptography techniques encrypt the GPS signals, which require a key to decrypt [4]. Techniques under the second category extract spatial and geometrical characteristics, or physical layer characteristics, such as angle-of-arrival, signal strength, signal phase, and discontinuities from legitime GPS signals. The third category is based on external UAV characteristics, such as speed and acceleration, that that can be measured by the sensors of the UAV flight control system, such as barometer, inertial measurement unit (IMU), and compass.

These GPS spoofing detection methods have some drawbacks that limit their application. For instance, cryptographic methods are not practical for civil applications as they require encryption/decryption keys (GPS signals have to be unencrypted for these applications). Methods based on signal processing or external characteristics of UAVs may require additional hardware (sensors or antenna) or auxiliary equipment, apply changes to the interface specifications, or need extensive signal processing capabilities, which adversely affect real-time system performance or require additional communication overhead.

Inertial navigation systems (INS) techniques require continuous inertial sensor calibration as the error in position estimates and their covariance continuously grow without bounds. For the detection of GPS spoofing attacks, these techniques can be only used when the quality of sensors with respect of size and cost is high. Therefore, using such detection techniques for small drones is not possible [5]. In addition, using sensors, such as gyroscope and accelerator, also involves some limitations in detecting GPS spoofing attacks. For example, the accelerator can only measure changes in the velocity [6]. Furthermore, this sensor cannot measure the rotation around its own axis of movement. Therefore, it has to be used with a gyroscope to measure angular velocities. The accelerator is also sensitive to temperature, which makes it difficult for it to perform properly in different environmental situations of UAVs. Moreover, Gyroscope is a sensor that does not measure linear motion in any direction or any static angle of orientation [7]. Therefore, as these two sensors are the two main components of INS and IMU-based techniques, and based on their drawbacks, they cannot be used in detecting GPS spoofing attacks on UAVs [8].

Several studies based on traditional machine learning (ML) techniques have been proposed to classify and detect GPS spoofing attacks on UAVs. Examples of such techniques include artificial neural networks [2] and tree models [9]. Such models provide effective solutions for detecting these in the detection of GPS spoofing. Such models provide effective solutions in the detection of GPS spoofing [10].

Ensemble learning techniques are considered as one of the main developments in machine learning in the past decade as they perform better than traditional machine learning methods [11]. Examples of ensemble models that have been proposed for detecting cyber-attacks are bagging, boosting, and stacking [12]. The stacking classifiers apply meta-learning algorithms to select the best combinations of the base machine learning algorithms. The bagging methods use a combination of repetitive techniques to generate several sets from the original data and evaluate the performance simultaneously. In Boosting algorithms, the weight of observation is adjusted based on the last classification. Therefore, these three ensemble techniques can provide a better performance than a single conventional machine learning (ML) model. However, these models deal with some limitations, such as the difficulty of interpreting outputs and low or high rates of bias, which may lead to under or over-fitting issues.

Therefore, a holistic solution that can easily interpret and perform better than any single conventional ML model in detecting GPS spoofing attacks is known as multiple classifier systems (MCS). In this technique, a pool of classifiers is competing to provide the best prediction for a data sample, and the final result belongs to the most efficient base classifier. One example of MCS approach is the dynamic classifier selection (DCS) [13], which focuses on learning methods that automatically choose a subset of techniques in the prediction process. DCS focuses on fitting several ML classifiers on a training dataset and choosing the model that provides the best result in the prediction process based on specific proposed factors.

In this work, we propose two dynamic-based selection methods that detect GPS spoofing attacks on UAVs: Metric-Optimized Dynamic (MOD) selection and Weighted-Metric-Optimized Dynamic (WMOD) selection. We implement ten well-known supervised machine learning classifiers in both the proposed methods. These models are Support Vector Machine, Naive Bayes, Decision Tree, K Nearest Neighbor, Linear Discriminative Analysis, Random Forest, Artificial Neural Network, Logistic Regression, Elastic Net, and AdaBoost. The two proposed classifier selection methods are trained and tested using a dataset with 13 GPS signals features built from real-time experiments and MATLAB attack simulations. The evaluation is conducted in terms of probability of detection ($P_{d}$), probability of false alarm ($P_{fa}$), probability of misdetection ($P_{md}$), accuracy( ACC), and processing time.

The contributions of this paper are:

- A one-stage ensemble feature selection technique to identify correlated and low importance features simultaneously.
- Two dynamic-based selection methods, MOD and WMOD, for efficient detection of spoofing signals.
- Performance comparison of the MOD and WMOD dynamic methods with bagging, boosting, and stacking-based ensemble models for validating the proposed techniques.

The remainder of this paper is organized as follows: Section 2 reviews the related works, while Section 3 illustrates the proposed architecture. Section 4 describes the materials applied in this work and highlights the methodology of the study. Section 5 discusses the simulation results. A conclusion is presented in Section 6.

## 2. Related Work

Several studies have been performed on GPS spoofing detection and mitigation methods. For instance, the authors of [7] proposed a GPS spoofing detection method that depends on the acceleration error calculated by estimating the acceleration from the GPS receiver and the acceleration measured from the IMU. In [9], the authors used IMU measurements (angle, velocity, and acceleration) and GPS data (longitude and latitude) in a two-step method that applies the XGBoost model and a Genetic Algorithm, to detect GPS-spoofing attacks. XGBoost was applied to learn the relationship between the IMU and GPS data, while the Genetic Algorithm was applied to tune the training parameters. An approach based on an artificial neural network was proposed in [2] to detect GPS spoofing signals. Several features, such as pseudo-range, doppler shift, and signal-to-noise ratio (SNR), were used to perform the GPS signal classification. Different neural network configurations were analyzed and tested. The proposed method revealed an acceptable efficiency in terms of probability of detection and probability of false alarm.

In [14], the authors proposed an anti-spoofing model that used linear regression to predict and model the optimal UAV route to its destination and used Long Short-Term Memory in the trajectory prediction. The model provides more than one detection scheme for GPS spoofing signals to improve UAV flight security and sensitivity to deception signal detection. Simulation experiments have determined that this method could enhance the ability to resist GPS spoofing without increasing hardware costs. Another GPS spoofing detection method was proposed in [15], based on the vision sensor combined with a UAV’s sensors, monocular camera, and IMU. This method used vision sensors combined with IMU data to detect GPS spoofing. Another vision-based UAV spoofing detection method that utilized Visual Odometry was presented in [16], which uses the UAV camera since fake GPS signals would not alter its images. The UAV relative trajectory can be extracted from images using Visual Odometry. This extracted trajectory is compared with flight trajectory information obtained from GPS positions, to detect the spoofed signals.

In [17], the authors proposed a GPS spoofing-detection framework that needs minimal prior configuration and applies information fusion. The real-time detection scheme derives the current UAV location from IMU and compares it to the location information received by the GPS receiver to determine if the UAV system was experiencing a GPS spoofing attack. In [18], the authors proposed a new algorithm to handle GPS spoofing attacks that caused unknown sudden system state variable changes. The compensation of the GPS spoofing effect was manipulated using a prediction discrepancy based on a particle filter algorithm. The proposed algorithm decreases the effects of GPS spoofing errors and estimates the true position of the UAV in the presence of GPS spoofing attacks. In [19], the authors proposed a spoofing detection and classification algorithm based on Least Absolute Shrinkage and Selection Operator. They used some signal processing techniques to observe the decomposition of two code-phase values for authentic and spoofed signals using a certain threshold to mitigate false alarms. The proposed method achieves a promising detection error rate for a spoofer attack in nominal signal-to-noise ratio conditions.

In [20], the authors proposed a methodology that consists of several ML models with a set of values for K-folds where voting techniques are integrated to choose the learning model that achieves the highest accuracy. In [21], a hardware-based solution was proposed to detect GPS spoofing attacks. The authors demonstrated a simple method to detect hijacking based on gyroscopes measurements and GPS data. A switching mode resilient detection and estimation framework for GPS spoofing attacks has been studied in [22]. The authors tried to address the sensor drift issue by keeping the estimation errors to remain in a tolerable region with high probability.

Machine learning methods do not require additional hardware, which may be attractive for small civilian UAVs. For instance, in [23], the authors proposed an approach to detect UAV GPS spoofing attacks based on the analysis of state estimation using Support Vector Machine. The proposed method detects GPS spoofing attacks to some extent; however, the system experienced performance degradation during long attacks due to the interaction with the GPS sensor, especially with the Micro-Electro-Mechanical Systems sensors. In [24], a GPS spoofing detection method was proposed that leverages the uplink received signal strength measurements collected from base stations to identify the adaptive trustable residence area, which represents the trust region within which the UAV GPS position should be located to be classified as authentic or non-spoofed. In [3], the authors proposed a method for GPS spoofing attack detection based on a machine learning algorithm, Long Short-Term Memory, and compared the results to a method based on specifically designed UAV flight paths. This method can detect attacks well when the flight trajectory is not complicated. Table 1 provides a summary of existing studies in literature with their advantages and limitations.

Although many spoofing detection techniques have been proposed in the literature, spoofers are continually evolving to produce new GPS spoofing attacks that are hard to detect, which increases the necessity to develop new mechanisms to prevent this kind of attack. Ensemble learning techniques can be a practical solution to address the limitations of the existing methods. In literature, there are no studies to investigate the performance of such approaches in detecting GPS spoofing attacks targeting UAVs; however, ensemble approaches, namely, bagging, boosting, and stacking, have been frequently utilized in detecting cyber-attacks in wireless communication systems. For instance, in [26], the authors proposed a stacked-based ensemble model to classify and detect attacks on wireless networks. The proposed approach consists of several base learning methods, namely, Support Vector Machine, Decision Tree, Random Forest, and Artificial Neural Network. The stacking approach outperforms the base learners. In [27], the authors compared different ensemble models, namely, bagging, boosting, and stacking, for predicting received signal power on UAVs. Their results demonstrate that the stacking model, including Support Vector Machine, Artificial Neural Network, and Gaussian Process, outperformed other base classifiers.

Dynamic classifier selection methods have been recently proposed as ensemble approaches that select the best performance ML model among all base models. To the best of our knowledge, no studies proposed such a technique for classifying and detecting GPS spoofing attacks on UAVs. Therefore, to fill the existing gap, two dynamic-based selection methods are proposed that use ten machine learning models. These methods select the ML method that provides best results to detect the presence or absence of an attack. To validate our proposed techniques and demonstrate that they provide optimal results, we compared our proposed methods with the three most known ensemble models, namely, bagging, boosting, and stacking, with our proposed techniques.

## 3. Proposed Architecture

**Figure 1:**
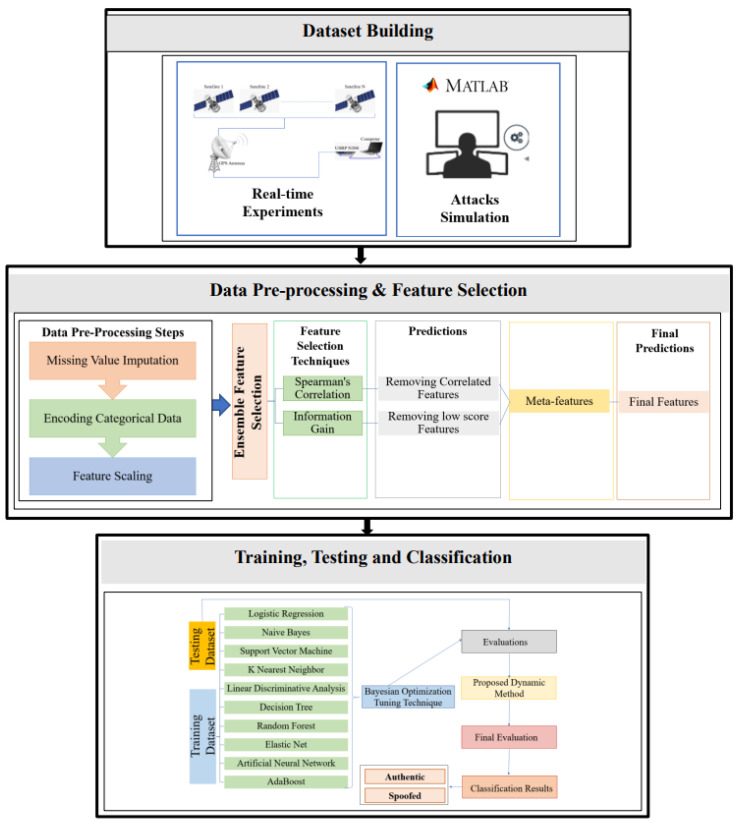
Overview of the Proposed Architecture.

The proposed system architecture is shown in Figure 1. This system consists of three phases: dataset building, data pre-processing and feature selection, and training and classification. For the dataset building, real-time experiments were conducted to collect real GPS signals, while attacks were generated through simulations. Features were identified and extracted from the real GPS signals, and the attack simulated signals [10]. The features for all samples are included in a dataset to be pre-processed. The second phase is the data pre-processing and feature selection, which focuses on missing value imputation, categorical data encoding, feature scaling, identifying correlated features, and discarding low importance features. In this study, we use feature scaling and transfer categorical feature values to numerical values to avoid any bias in the corresponding dataset.

Two feature selection techniques are applied: Spearman Correlation and Information Gain. The ensemble feature selection can simultaneously identify the correlated and low importance features and discard them from the corresponding dataset [28]. The primary aim of using ensemble feature selection is to decrease the dimensionality of the dataset and identify the most important features [29] that can enhance the performance of the proposed model.

For the training, testing, and classification phases, we implement ten traditional ML techniques: Support Vector Machine, Naive Bayes, Decision Tree, K Nearest Neighbor, Linear Discriminative Analysis, Random Forest, Artificial Neural Network, Logistic Regression, Elastic Net, and AdaBoost. To get the optimal results of each model, a hyperparameter tuning technique, Bayesian optimization, is used.

Two dynamic methods are implemented for detecting GPS spoofing attacks targeting UAVs. The proposed methods dynamically choose the classifier that achieves the best results for the considered performance metrics. Incoming signals are classified as authentic or spoofed in the prediction phase, and their probabilities is evaluated.

## 4. Methodology

In the following, we discuss the dataset, data pre-processing, feature selection, description of the proposed models, and hyperparameter tuning that are used in this study, as follows:

### 4.1. Dataset

In this study, the used dataset was built in the work described was implemented in [10]. Real-time experiments and simulations were conducted to collect a dataset of authentic and spoofed signals at different dates in several locations. The hardware used in the implementation consisted of a universal software radio peripheral (USRP), a front-end active GPS antenna, and an I5-4300U laptop with 8 G RAM running with Ubuntu 16.04.7 LTS version. GPS attacks were simulated using MATLAB by considering three types of spoofing attacks with different complexity levels: simplistic, intermediate, and sophisticated. Each of these attacks impacts specific features of the GPS signals, such as Doppler Shift Measurement, Receiver Time, and Pseudo Range. In simplistic spoofing attacks, a fake GPS signals, which was unsynchronized with the authentic signals, was generated. In this case, higher Doppler Shift measurements were out of the normal range of ±20 Hz, leading to a signal drift. In this type of attacs, GPS spoofing signals are also transmitted at a higher power level, compared to that of authentic GPS signals, resulting in a higher Signal-to-Noise Ratio value.

In intermediate spoofing attacks, the attacker has a knowledge of UAV position. The intermediate attacker is able to control of the generated GPS signals. In this type of attack, the Doppler Shift Measurements and Pseudo Range values are kept within the normal ranges. In sophisticated attacks, the spoofer gains control over several channels of multiple synchronized antennas. This type of attack is the most threatening spoofing attack, due to the effect of multipath signals and the motion of the satellites and receiver.

**Figure 2:**
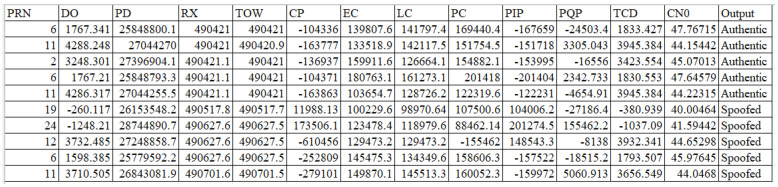
Sample of Dataset.

Thirteen features were extracted from various receiver stages, starting from the tracking loop to the observable block. The extracted features from the received GPS signals with their short descriptions are listed in Table 2. The corresponding dataset is balanced and contains 10,055 samples, of which 5028 are authentic signals and the remaining are equally divided between the three types of GPS spoofing attack signals. A sample of dataset is presented in Figure 2.

### 4.2. Data Pre-Processing

The dataset was previously pre-processed by identifying and removing any null, unknown, and noisy values during the missing value identification step [30]. The next step is to encode any categorical values to numerical values. There are only two categorical data values in our dataset, which represent the signals as attack or normal. For this purpose, we encode normal signals as 0 and spoofed signals as 1. Afterward, feature scaling is performed by applying normalization and standardization methods. Normalization can re-scale the values into ranges between 0 and 1. In this study, we use the power transformer technique based on the Yeo-Johnson transformer. Unlike other techniques, this method can handle positive, negative, and zero data values. We also applied a simple standardization technique, which re-scaled the values to a mean of 0 and a standard deviation of 1.

### 4.3. Feature Selection

Ensemble feature selection techniques are widely used to enhance the robustness of feature selection techniques. These techniques are classified into two categories, namely, homogeneous and heterogeneous. In homogeneous ensemble feature selection, the same method is used with different sizes of training data, while heterogeneous ensemble feature selection mostly focuses on different feature selection methods with similar training datasets. This study employs a heterogeneous ensemble feature selection technique using two traditional feature selection techniques, namely, Spearman’s Correlation and Information Gain. The goal of selecting these two feature-selection techniques is to remove correlated and unimportant features from the given dataset.

Spearman Correlation [31] primarily calculates the association and direction between each two features by calculating the score τ given by:

(1) $$\tau =1-\frac{6\sum_{i=1}^{n}{\left(d_{i}\right)}^{2}}{n(n^{2}-1)}$$

where $d_{i}$ is the difference between the two ranks of each observation, *i* is the index of the observation, and *n* is the number of observations. A feature is correlated if it attains a coefficient over 0.9. We consequently removed a feature from each pair of correlated features.

We also used the information-gain feature-selection technique, called mutual information [32], for feature importance to estimate the gain of each variable in terms of the target variable. The information gain, also known as entropy, is calculated for every feature; features with high entropy are selected as important features, and those with low entropy values are considered of low importance. Any feature that achieves an entropy less than 0.1 is discarded from the dataset in this work.

### 4.4. Hyperparameter Tuning

Several types of tuning techniques have been proposed in the literature; however, Bayesian optimization has emerged as an effective approach, outperforming other techniques such as random search and grid search since grid search suffers from the curse of dimensionality and random search is not suitable for training complex models [33,34,35]. Bayesian optimization can provide a practical solution to optimize functions using a computationally cheap surrogate model [36]. This approach can offer robust solutions for optimizing the black-box functions, applying a non-parametric Gaussian process to simulate unknown functions. A surrogate utility function, also known as the acquisition function, is another main component of Bayesian optimization, which is defined as a way to improve the optimality of the underlying function [37]. In this study, considering the benefits of Bayesian Optimization and shortcomings of other techniques, we employ this technique for optimization tuning.

### 4.5. Description of the Proposed Methods

Dynamic classifier selection techniques consist of a pool of homogenous or heterogeneous base classifiers. Homogenous classifiers are defined as using a set of classifiers that are of the same type built upon various data. In contrast, heterogeneous classifiers are designed using a group of classifiers belonging to various types built upon same data. In this work, we employed a set of heterogeneous base classifiers: Support Vector Machine (SVM), Naive Bayes (NB), Decision Tree (DT), K Nearest Neighbor (KNN), Linear Discriminative Analysis (LDA), Random Forest (RF), Artificial Neural Network (ANN), Logistic Regression (LR), Elastic Net (EN), and AdaBoost. The primary reason behind selecting heterogeneous classifiers is to increase the final model diversity without changing any model parameters [38].

**Figure 3:**
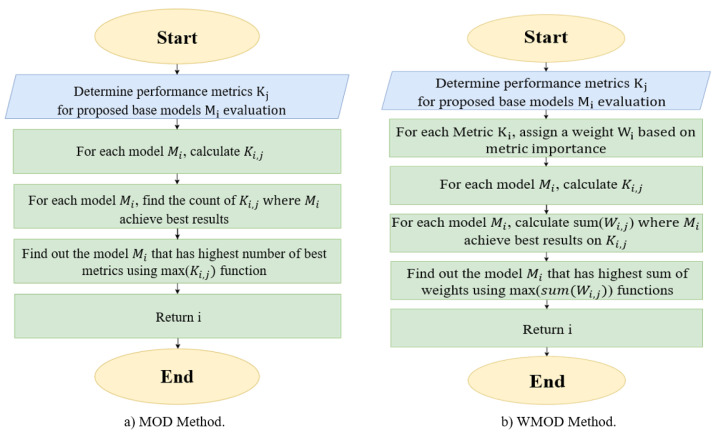
Flowcharts of the Proposed Dynamic Selection Methods.

We propose two dynamic selection methods for detecting GPS spoofing attacks on UAVs: MOD and WMOD classifiers, as shown in Figure 3. These methods focus on evaluating the ML models in terms of the probability of detection $P_{d}$, probability of false alarm $P_{fa}$, probability of misdetection $P_{md}$, and accuracy ACC. Figure 3a depicts the M base models and K performance metrics, i represents the base model index, and j represents the performance metric index. We initially calculate the performance metrics $\left(K_{i,j}\right)$ for every base model $M_{i}$ to find the optimal results; then, we determine the count $\left(K_{i,j}\right)$ where $M_{i}$ achieves the best results for every base model $M_{i}$. As a model achieves higher $P_{d}$ and ACC values and lower $P_{fa}$ and $P_{md}$ values, the model is considered better at detecting GPS spoofing attacks; therefore, the model with higher $P_{d}$ and ACC, and lower $P_{md}$ and $P_{fa}$, will be selected for the final incoming GPS spoofing signal detection. This concept is implemented in the proposed MOD classifier approach since the algorithm will identify the model with the highest number of best metrics, using Max($K_{i,j}$) for final detection.

MOD classifier is simpler to implement compared to other approaches, does not need extensive processing, does not require additional hardware, and has low time complexity. This classifier heavily depends on the selected base algorithm, which achieves the best metrics. As a result, no additional cost of processing or computational complexity was added to the overall algorithm. However, if two base models achieve the same number of best metrics, the MOD classifier will select one of the two classifiers trivially as the best model, in some cases when two base models achieve the same number of best metrics. To address this issue, we propose another approach: WMOD classifier. WMOD classifier, shown in Figure 3b, assigns a weight, w, for each performance metric to calculate a score using sum $\left(w_{i,j}\right)$ for each base model. In this model, a weight is assigned to each of the selected metrics based on their importance. We consider the importance of accuracy higher than the importance of $P_{d}$, $P_{fa}$, and $P_{md}$. Therefore, we assign a weight of 0.4 for accuracy, while other metrics, such as $P_{d}$, $P_{fa}$, and $P_{md}$, have each a weight of 0.2. We have to determine the count $\left(K_{i,j}\right)$ for every model $M_{i}$, where $M_{i}$ obtains the highest weights according to the defined weights. The model that achieves the best score is used for the final detection of any incoming signal.

## 5. Results

We ran our simulation on intel core i7-10750H, CPU of 2.60 GHz, and 16.0 GB memory. We used four evaluation metrics to assess the proposed model’s efficiency: the probability of detection $\left(P_{d}\right)$, probability of false alarm $\left(P_{fa}\right)$, probability of misdetection $\left(P_{md}\right)$, accuracy *(ACC)*, and processing time. These metrics were calculated using the following equations:

(2) $$P_{d}=\frac{T_{P}}{T_{P}+F_{N}}$$

(3) $$P_{fa}=\frac{F_{P}}{T_{F}+F_{N}}$$

(4) $$P_{md}=\frac{F_{N}}{T_{N}+F_{P}}$$

(5) $$ACC=\frac{T_{P}+T_{N}}{T_{P}+T_{N}+F_{P}+F_{N}}$$

where $T_{P}$ is the number of corrected predicted malicious flows, $T_{N}$ is the number of predicted normal flows, $F_{P}$ is the number of incorrectly predicted malicious flows, and $F_{N}$ is the number of incorrectly predicted normal flows.The processing time is defined as a time to train and test the classifiers. This metric highly depends on the ML model and dataset size. A three-fold cross-validation technique was applied to train 80% of the data and test 20% of the remaining dataset. The simulation analysis for the proposed dynamic methods was compared with the ten base selected classifiers in terms of the selected evaluation metrics.

**Figure 4:**
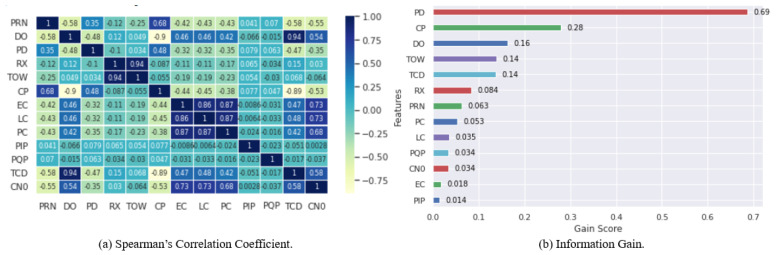
Importance of Features based on Ensemble Feature Selection: Spearman’s Correlation Coefficient and Information Gain.

Figure 4a,b show the results of the ensemble feature selection techniques, Spearman’s Correlation and Information Gain, respectively. We discarded one feature of each pair of correlated features with a mutual coefficient > 0.9 as well as features of low importance with scores < 0.1 from the corresponding dataset. As one can see in Figure 4a, two pairs of features have a high correlation; the first pair, *DO* and *TCD*, has a correlation of 95%, and the second pair, TOW and RX, has a correlation of 94%. In addition, *DO* has a higher importance than *TCD*, and TOW has a higher importance than *RX*. Therefore, *TCD* and *RX* were discarded from the dataset. The remaining features are the relevant features selected to classify GPS signals.

Table 3 provides the parameter setting and best parameter results obtained after applying the Bayesian Optimization algorithm. As can be seen, we specify several parameter settings with multiple values to check the optimality of every ML model. The parameter setting for every ML model is selected based on the provided values in Scikit-learn. Scikit-learn tool is a simple and efficient library that provides the suitable implementation for training, testing, and validating ML models, along with parameter settings for every ML model. The lists of setting parameters are provided in the table. These values are applied to achieve the best performance for each of the individual models. For instance, the activation function in the NN model is set to identity, logistic, tanh, or relu, and our selected tuning technique identifies Tanh as the activation function that guarantees the highest performance for the NN model. The NN model also has other parameters, including solver and alpha, that are required to be provided by tuning technique to achieve optimal results. In addition, the NB model consists of a parameter, namely, var_smoothing. This parameter is set to several values, provided in Scikit-learn tool. The Bayesian optimization technique identifies 1 $\times \;10^{-3}$ as a hyperparameter, among other values, that ensures the best possible performance for the NB model. The same observations can be seen for the other selected models. To this end, these best parameters are used in training the selected models to ensure the optimality of the results.

Ten ML models are used with their best parameters’ values in implementing the proposed dynamic classifiers. WMOD is proposed to handle a limitation of MOD. Such limitation occurs when two ML classifiers have the same number of metrics with the best results; therefore, two classifiers are selected as optimal. To address this issue, WMOD is proposed to return only the model with the best metric results.

**Figure 5:**
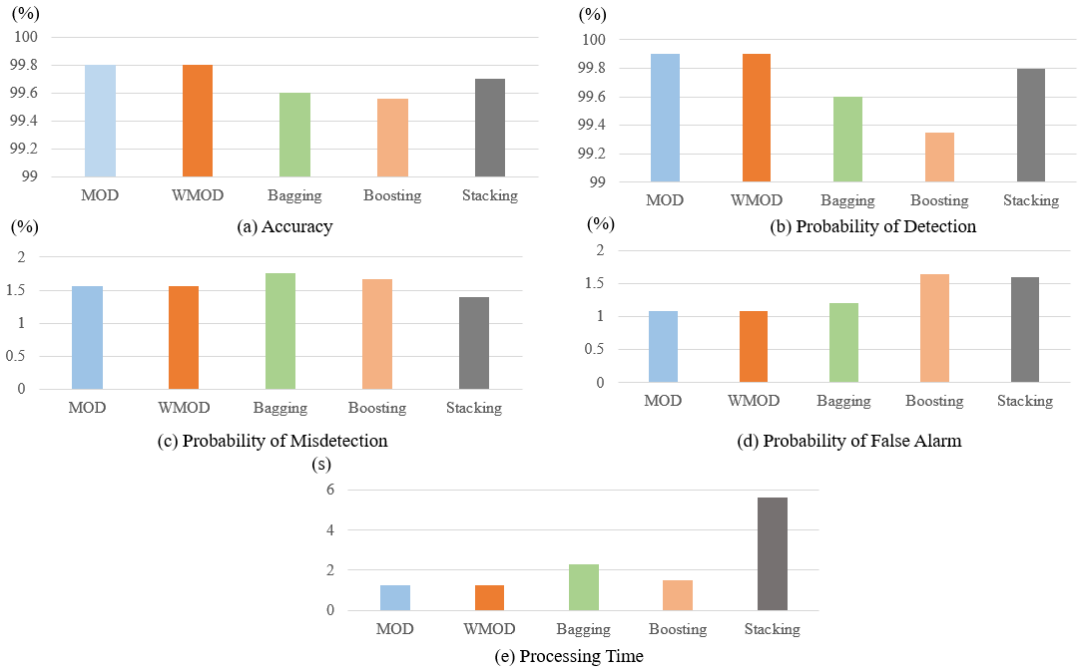
Evaluation results of the selected methods in terms of accuracy, probability of detection, probability of misdetection, probability of false alarm, and processing time.

Figure 5a provides the results of the proposed methods and the three ensemble models in terms of accuracy. As one can observe, the proposed MOD and WMOD dynamic methods provide the best results in terms of accuracy in comparison with bagging, boosting, and stacking-based ensemble models. As shown, the MOD and WMOD classifiers both have an accuracy of 99.8%. The stacking classifier has an accuracy of 99.7%, followed by bagging and boosting classifiers. Bagging model has an accuracy of 99.6%, while the boosting-based ensemble has the lowest accuracy of 99.56% compared to the other classifiers.

Figure 5b presents the results of the five models in terms of probability of detection. As can be seen, the proposed dynamic methods outperform the three ensemble models in terms of probability of detection with a slight difference. MOD and WMOD have the highest probability of detection of 99.9%, followed by the stacking, bagging, and boosting models. The stacking model has a probability of detection of 99.8%, bagging model has a probability of detection of 99.6%, and the boosting model has the lowest probability of detection of 99.35%.

Figure 5c illustrates the results of the proposed dynamic selection methods and the three ensemble models in terms of the probability of misdetection. As one can observe, MOD and WMOD have an acceptable probability of misdetection; however, the lowest probability of misdetection belongs to the stacking model. The proposed dynamic selection methods have a probability of misdetection of 1.56%, while the stacking model has a probability of misdetection of 1.4%. The other two ensemble models, bagging and boosting, also provide acceptable probability of misdetection results of 1.67% and 1.76%, respectively.

Figure 5d provides the results of the 5 models in terms of the probability of false alarm. As can be seen, MOD and WMOD have the lowest and best probability of false alarm compared to the other ensemble models. These methods have a probability of false alarm of 1.09%, the bagging classifier has a probability of false alarm of 1.2%, and the stacking and boosting have a probability of false alarm of 1.6% and 1.64%, respectively.

Figure 5e provides the results of the selected models in terms of their processing time. As one can see, the MOD and WMOD classifiers require a processing time of 1.24 s, which is considered much lower in comparison with other techniques, such as bagging, boosting, and stacking. The bagging classifier has a processing time of 1.321 s, while the boosting classifier achieves a processing time of 1.987 s. The stacking classifier has a processing time of 5.432 s, which is significantly higher than MOD and WMOD.

**Figure 6:**
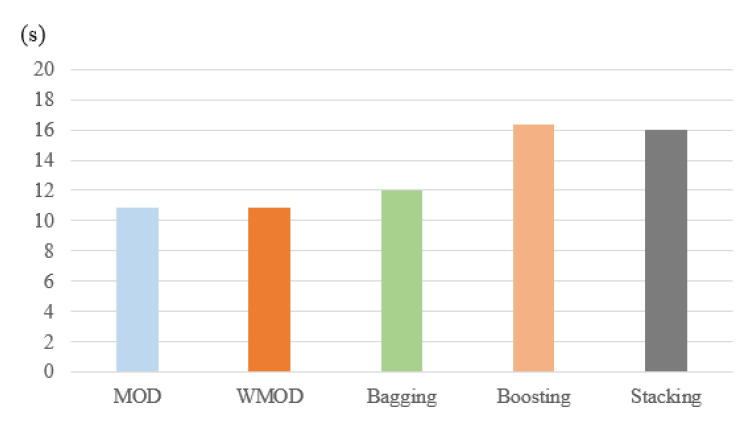
Number of False Positives for the Proposed Dynamic Selection Methods: MOD and WMOD Against the Classical Ensemble Techniques.

The number of false positives ($f_{p}$) is another important factor in evaluating models that compares the number of false positively predicted samples to total number of samples that are negatively predicted. Figure 6 provides the number of false positive for the highlighted methods. As one can observe, the MOD and WMOD provides the best number of false positives, followed by bagging, stacking, and boosting. The proposed dynamic selection methods have a number of false positives of 10.9 per second. In contrast, the bagging classifier has a number of false positives of 12 per secondm and the stacking and boosting classifiers have a number of false positives of 16 and 16.4 per second.

Table 4 provides the results of the proposed dynamic selection methods and the ensemble techniques, namely, bagging, boosting, and stacking. This table shows that MOD and WMOD have the best results in terms of accuracy, probability of detection, and probability of misdetection. In contrast, the stacking model provides the best result in terms of the probability of misdetection. It can be noticed that the proposed methods, MOD and WMOD, provide a probability of misdetection of 1.56%, which is higher than the stacking model by 0.16% considered as an insignificant difference. In contrast, the stacking model has a probability of false alarm of 1.6%, which is 0.51% higher than the probability of false alarm of these proposed methods. In addition, this stacking model has an accuracy of 99.7% and a probability of detection of 99.8%, which are 0.1% lower than the accuracy and probability of detection of the proposed dynamic selection methods.

As one can observe, the processing time of the proposed classifiers is 1.24 s, which is significantly lower than that of the other ensemble approaches. The bagging classifier has a processing time of 2.321 s, which is 1.081 s higher than that of MOD and WMOD. The boosting classifier has a processing time of 1.511 s, which is 0.271 s higher than that of the proposed classifiers. The stacking classifier has the worst processing time, which is 4.41 s higher than the processing time of MOD and WMOD.

To shed more light on the effectiveness of the proposed methods, we calculate the number of false positives ($f_{p}$) for MOD and WMOD, followed by bagging, stacking, and boosting. The proposed methods provide a number of false positives of 10.9 per second, which is 1.1 lower than the bagging classifier, 5.5 lower than the boosting classifier, and 5.1 lower than the stacking classifier. To conclude, our proposed classifiers provide higher accuracy and probability of detection, and lower probability of misdetection, false alarm processing time, and the number of false alarms compared to the other classical ensemble techniques.

In short, the key insights can be summarized as follows:

- Ensemble feature selection removes the correlated and low importance features and decreases computational power and time.
- MOD and WMOD methods dynamically select one best classifier between the implemented ML models.
- The proposed dynamic methods can choose the best metric among the implemented models based on the considered metrics, which means such methods can be easily extended to include additional metrics that can significantly enhance the selection criteria.
- Comparison of the ensemble models with the proposed dynamic methods shows that the two dynamic methods can achieve good results in detecting GPS spoofing attacks on UAVs.

## 6. Conclusions

Interest in detecting GPS spoofing attacks on UAVs has increased significantly in the last decade, leading to considerable progress in different technologies. Several techniques have been proposed to identify and detect these vulnerabilities; however, this field of study still needs to address several challenges and limitations, such as high misdetection and false alarm rates. In this work, we used a one-stage heterogeneous ensemble feature selection to discard correlated and low importance features from the considered dataset using Spearman Correlation and Information Gain. As a result, two features, RX and TCD, were discarded from the given dataset. We implemented two dynamic selection methods, MOD and WMOD, which dynamically selected the best ML model among the ten implemented. However, MOD has a limitation when two ML classifiers have the same number of metrics with the best results. WMOD addresses this limitation and perfectly optimizes the selection criteria. The results show that MOD and WMOD have an accuracy of 99.6%, a probability of detection of 98.9%, a probability of false alarm of 1.56%, a probability of misdetection of 1.09%, and a processing time of 1.24%. These results outperform those of the existing ensemble learning models.

## Figures and Tables

**Table 1:** Existing Literature on Detecting GPS Spoofing on UAVs.

Category	Approach	Advantages	Limitations
External UAV characteristics	Acceleration error [7]	• Uses magnitude acceleration error to provide better performance	• Depends on accelerator error. • Pre-defined probability of false alarm.
	IMU-based [9]	• Provides a detection rate of 96.3% and 100% in hijacked and non-hijacked cases.	• Only detects attacks with similar behaviors during training.
	IMU-based [25]	• In best cases, detection rate of 98.6%,within 8 s when the system is under attack. • Provides a precision of 97%, a recall of 97%, and F1-score of 97%.	• In worse cases, the detection of GPS spoofing attacks can table over 28 s after the UAV started its mission.
	Gyroscopes measurement-based [21]	• Easy to be implemented in any drone.	• Needs motion sensors (gyro-scopes and accelerators for detection. These are power hungry.
Artificial Intelligence Method	Artificial Neural Network-based [2]	• Provides an accuracy of 98.3%, a probability of detection of 99.2%, a probability of misdetection of 2.6%, and probability of false alarm of 0.8%.	• Uses a dataset with only 5 features and very limited samples.
	Linear regression-based and long short term memory [14]	• Works effectively in a case of UAV flying along the specified rout.	• Lack of optimization methods from the perspective of UAV sensor integrated navigation and UAV attitude control.
	Prediction-discrepancy based [18]	• Reduces the effects of GPS spoofing errors and estimates the true position of the UAV in the presence of GPS spoofing attacks.	• Evaluated only based on accuracy and redundancy.
	Least Absolute Shrinkage and Selection Operator [19]	• Provides a 0.3% detection error rate for a spoofing attack in nominal signal-to-noise ratio conditions and an authentic-over-spoofer power of 3 dB.	• Uses a public old dataset, namely, Texas spoofing test battery as benchmark. • Lack of using common evaluation metrics, such as the probability of misdetection.
	K-learning based [20]	• Provides an accuracy of 99%, a precision of 98%, a recall of 99%, and F-score of 98%.	• Uses only Shimmer and Jitter as features in the dataset.
	Resilient State Estimation [22]	• Addresses the sensor drift problem.	• Evaluated only based on estimated error, and statistics of attacks.
	Support Vector machine [23]	• Improves the performance in case of using magnetometer sensors.	• Performance degradation during long attacks.
	5G-assisted position monitoring [24]	• A detection rate of 95%, and F1-score of 88%.	• Lacks of several evaluation metrics.
	Long-Short Term Memory [3]	• A comprehensive comparison with encryption-based detection techniques in terms of detection rate and time cost.	• Detection rate of 78% and a time cost of 3s. • Detection rate is high when the flight trajectory is not complicated.
Signal Processing	Vision-based [15]	• Detects spoofing attacks with an average of 5s based on several parameters.	• Only applied when the attacker is visible.
	Vision-based [16]	• Detects spoofing in the long-range UAV flights when the changes in UAV flight direction is larger than 3° and in the incremental UAV spoofing with the redirection rate of 1°.	• Only applied when the attacker is visible.

**Table 2:** List of Features.

Feature	Abbreviation	Description
Satellite Vehicle Number	PRN	Identifying uniquely each satellite in orbit.
Doppler Shift Measurement	DO	Difference in the frequency of a GPS receiver moving relatively to its source. Difference in the frequency of a GPS receiver moving relatively to its source.
Pseudo Range	PD	Difference between the transmission and the reception time.
Receiver Time	RX	Time of transmission of the navigation messages.
Decoded Time Information	TOW	Information regarding the reception time of a subframe.
Carrier Phase Shift	CP	Beat frequency difference between the received carrier and a receiver-generated carrier replica.
Prompt Correlator	PC	Happens when the replica signal generated from the receiver is compatible with the incoming signals.
Late Correlator Output	LC	Occurrs at the 1/2 chip spacing after the prompt correlator.
Early Correlator Output	EC	Happens at the 1/2 chip spacing before the prompt correlator.
Prompt In-phase Prompt	PIP	In-phase component of the Prompt correlator amplitude.
Prompt Quadrature Prompt	PQP	Quadrature component of the prompt correlator amplitude.
Carrier Loop Doppler Measurements	TCD	Doppler shift that is measured during the correlation stage.
Signal to Noise Ratio	CN0	Doppler shift that is measured during the correlation stage. Ratio of the power signal to noise.

**Table 3:** Parameter Setting Results.

Model	Parameter Setting	Best Parameters
SVM	C = [0.1, 1, 10, 100],	C = 10,
	degree = [1, 2, 3, 4, 5],	degree = 5,
	gamma = [1, 0.1, 0.01, 0.001, 0.0001].	gamma = 0.1.
NB	var_smoothing = [1e-2, 1e-3, 1e-4, 1e-5, 1e-6, 1e-7, 1e-8, 1e-9, 1e-10, 1e-11, 1e-12, 1e-13, 1e-14, 1e-15].	var_smoothing = 1e-3.
DT	Criterion = [‘gini’, ‘entropy’],	criterion = ‘entropy’,
	Splitter = [‘best’, ‘random’],	splitter = ‘best’,
	max_features = [‘auto’, ‘sqrt’, ‘log2’],	max_features = ‘auto’,
	max_depth = range (1, 32).	max_depth = 26.0.
RF	n_estimators = [10, 100, 1000, 10,000],	n_estimators = 1000,
	max_depth = range (10, 200),	max depth = 110,
	min_samples_split = range (2, 10).	min_samples_split = 2.
KNN	n_neighbors = range (1, 20),	n_neighbors = 6,
	p = range (1, 10).	p = 1.0.
LDA	Solver = [‘svd’,‘lsqr’].	solver = ‘lsqr’.
NN	Activation = [‘identity’, ‘logistic’, ‘tanh’, ‘relu’],	activation = ‘tanh’,
	Solver = [‘lbfgs’, ‘sgd’, ‘adam’],	solver = ‘lbfgs’
	Alpha = linspace(0.0001, 0.5, num = 50).	alpha = 0.0409,
LR	l1_ratio = linspace(0.0001, 1, num = 50),	l1_ratio = 0.0001,
	C = [0.1, 1, 10, 100],	C = 100.0,
	Solver = [‘newton-cg’, ‘sag’, ‘lbfgs’].	solver = ‘lbfgs’.
EN	l1_ratio = linspace(0.0001, 1, num = 50),	l1_ratio = 0.190,
	alpha = linspace(0.0001, 2, num = 50),	alpha = 0.1409,
	selection = [“random", “cyclic"].	selection = ‘cyclic’.
AD	n_estimators = [10, 100, 1000, 10,000].	n_estimators = 100.

**Table 4:** Evaluation Results of the Proposed Dynamic Selection Methods and Ensemble Models.

Methods	Metrics					
	***ACC*** (%)	$P_{d}$ (%)	$P_{\mathrm{md}}$ (%)	$P_{\mathrm{fa}}$ (%)	Processing Time (s)	$f_{p}$ (s)
MOD	99.8	99.9	1.56	1.09	1.24	10.9
WMOD	99.8	99.9	1.56	1.09	1.24	10.9
Bagging	99.6	99.6	1.76	1.2	2.321	12
Boosting	99.56	99.35	1.67	1.64	1.511	16.4
Stacking	99.7	99.8	1.4	1.6	5.65	16

## Data Availability

Dataset will be available in Github and other sites.
